# Midwife-performed checklist and ultrasound to identify obstetric conditions at labour triage in Uganda: A quasi-experimental study

**DOI:** 10.1016/j.midw.2021.102949

**Published:** 2021-05

**Authors:** Jude Mulowooza, Nicole Santos, Nathan Isabirye, Innocent Inhensiko, Nancy L. Sloan, Sachita Shah, Elizabeth Butrick, Peter Waiswa, Dilys Walker

**Affiliations:** a Makerere University, School of Public Health, P.O Box 7072, Kampala, Uganda; b Institute for Global Health Sciences, University of California San Francisco, 550 16th Street, 3rd floor, San Francisco, CA 94158 United States; c Department of Emergency Medicine, University of Washington, 325 9th Ave., Seattle, WA 98104 United States; d Global Health Department of Public Health Sciences, Karolinska Institutet, Stockholm, Sweden; e Department of Obstetrics, Gynaecology and Reproductive Sciences, University of California San Francisco, United States

**Keywords:** Labour triage, Obstetric ultrasound, Checklist, Midwife

## Abstract

**Objective:**

The aim of this study was to evaluate the effect of a midwife-performed checklist and limited obstetric ultrasound on sensitivity and positive predictive value for a composite outcome comprising multiple gestation, placenta praevia, oligohydramnios, preterm birth, malpresentation, abnormal foetal heart rate.

**Design:**

Quasi-experimental pre-post intervention study.

**Setting:**

Maternity unit at a district hospital in Eastern Uganda.

**Interventions:**

Interventions were implemented in a phased approach: standardised labour triage documentation (Phase 1), a triage checklist (Phase 2), and checklist plus limited obstetric ultrasound (Phase 3).

**Participants:**

Consenting women presenting to labour triage for admission after 28 weeks of gestation between February 2018 and June 2019 were eligible. Women not in labour or those requiring immediate care were excluded. 3,865 women and 3,937 newborns with similar sample sizes per phase were included in the analysis.

**Measurement and findings:**

Outcome data after birth were used to determine true presence of a complication, while intake and checklist data were used to inform diagnosis before birth. Compared to Phase 1, Phase 2 and 3 interventions improved sensitivity (Phase 1: 47%, Phase 2: 68.8%, Phase 3: 73.5%; *p* ≤ 0.001) and reduced positive predictive value (65.9%, 55%, 48.7%, *p* ≤ 0.001) for the composite outcome. No phase differences in adverse maternal or foetal outcomes were observed.

**Conclusion:**

Both a triage checklist and a checklist plus limited obstetric ultrasound improved accurate identification of cases with some increase in false positive diagnosis. These interventions may be beneficial in a resource-limited maternity triage setting to improve midwives' diagnoses and clinical decision-making.

## Introduction

In 2017, Sub-Saharan Africa had the largest number of maternal and under-five deaths globally with 196,000 and 2.95 million deaths, respectively (UNFPA, 2019; Liu et al., 2016). Some of the maternal and perinatal conditions that contribute to this burden, such as antepartum haemorrhage, hypertension, preterm complications and intrapartum-related events, can be mitigated by timely and accurate identification and management of pregnancy complications (WHO, 2016). This relies dually on adequate antenatal care (ANC) and skilled attendance at birth. Despite an increase in facility-based births in many low- and middle-income countries (LMICs), quality of care at the time of labour and birth is impeded by limited uptake of evidence-based practices, late recognition of complications, and delays in receiving care (Bhutta et al., 2014; Goodman et al., 2017). Furthermore, coverage of four or more ANC visits and quality of ANC content varies in such settings (Benova et al., 2018). In Uganda, for example, 60% of 15–49 year old women received four ANC visits in 2011 and the median gestational age for ANC initiation was almost 4.7 months (Uganda DHS, 2016). The World Health Organisation (WHO) now recommends an 8-contact ANC schedule and that women receive at least one ultrasound scan before 24 weeks of gestation to ascertain gestational age, multiple gestation, and congenital abnormalities (WHO, 2016). However, the proportion of women who achieve either of these recommendations are unknown.

Careful and accurate assessment when a woman first presents in labour (i.e., labour triage) can help providers identify pregnancy complications prior to birth and prioritise appropriate care, particularly amongst women who received inadequate ANC and have undetected conditions (Sandy et al., 2016; Rashidi Fakari et al., 2019a). In many LMICs, standardised guidelines and interventions at this initial assessment point are often lacking (Baker, 2009; Rashidi-Fakari et al., 2019b). At Mulago National Hospital in Uganda, for example, less than half of women who presented for maternity admission were triaged in terms of referral status, prior Caesarean section or bleeding (Forshaw et al., 2016). However, a recent study at a large referral hospital in Ghana demonstrated that specialised triage training, introduction of a standardised labour triage screening tool and processes led to reductions in patient waiting time and improved documentation of care plans (Floyd et al., 2018; Goodman et al., 2018). Moreover, intrapartum obstetric ultrasound administered in high-income triage contexts has been shown to reliably detect multiple gestation, placenta location, fluid volume, presentation, and foetal cardiac activity (Gabbe et al., 2016; Wiafe et al., 2016). In LMICs, intermittent auscultation of foetal heart rate (FHR) through an ultrasound device has low-certainty evidence for improved detection of FHR abnormalities compared to a Pinard stethoscope (Bricker et al., 2015).

Further investigation of interventions to effectively screen women at labour triage in resource-limited contexts are needed to improve identification and management of pregnancy complications. Therefore, this study evaluated if a triage checklist and limited obstetric ultrasound administered by midwives can be implemented at a district hospital in Eastern Uganda to improve diagnosis of select maternal and foetal conditions, including preterm labour, multiple gestation, oligohydramnios, placenta praevia, malpresentation, and abnormal FHR.

## Methods

### Study design

This quasi-experimental study compared the phased implementation of three interventions between February 2018 and June 2019: Phase 1 introduced standardised intake/outcome documentation; Phase 2 included standardised documentation plus a triage checklist; Phase 3 included standardised documentation, a triage checklist plus limited obstetric ultrasound. The phased-in design assessed the added value of incremental package components (Phase 2 and 3) compared to the referent period (Phase 1).

### Setting

The study was conducted at a district hospital in Eastern Uganda which has an estimated 7600 deliveries annually. It serves a catchment area of six districts (approximately 0.5 million people) and receives referrals from public health centres in the area. The maternity ward has 17 midwives on staff; each shift is staffed with approximately four midwives, one obstetrician/gynaecologist and one medical officer. The hospital's Caesarean rate was 25% in 2017 according to District Health Information Software (DHIS-2) data (Uganda Ministry of Health, 2017).

### Outcomes

Our primary objective was to determine if a clinical assessment checklist alone (Phase 2) or checklist with limited obstetric ultrasound (Phase 3) at labour triage could improve diagnostic accuracy of a composite variable comprising preterm labour, multiple gestation, oligohydramnios, placenta praevia, malpresentation, and abnormal FHR. We also assessed any maternal condition (multiple gestation, oligohydramnios, placenta praevia and preterm birth) and any foetal condition (malpresentation, abnormal FHR).

For diagnostic measures, we ascertained sensitivity, the proportion of outcome-defined cases who were identified at intake correctly, and positive predictive value (PPV), the proportion of those screened positive who were true cases. Outcome data collected after birth were used to classify the true presence of a complication, while intake and checklist data were used to inform intake diagnosis before birth. An example of correct identification is if twins were born and the mother was diagnosed with a multiple gestation on admission.

### Interventions

Each intervention phase began with a training and pilot period. All clinical activities were conducted by midwives who were trained in study procedures since the goal was to determine if a checklist and/or limited obstetric ultrasound could be performed without the addition of extraneous personnel. In most cases, the midwife who conducted the clinical intake assessment was involved in the clinical decision-making for birth, consulting the hospital's obstetrician/gynaecologist or medical officers when needed. Non-clinical activities, such as consenting procedures and data abstraction, were done by study-hired research nurses. Each shift was covered by at least one study-trained midwife and one research nurse.

Phase 1 (February - May 2018) introduced an intake log with a single record for each eligible patient assessed in triage. This documented participant demographic information, gestational age at presentation, and clinical diagnoses made before decision to admit, send home or refer. Phase 1 captured baseline data and supplemented the existing intake system information. The intake log did not include any clinical prompts related to the conditions of interest, as this phase was meant to capture midwives' clinical assessments using the existing standard of care. During a 5-day orientation workshop and piloting phase, three midwives and two research study nurses were trained on Phase 1 study procedures and protocols.

Phase 2 (June - September 2018) introduced a triage checklist. This checklist was created by the study team which included two U.S.-based obstetricians and the study hospital's obstetrician/ gynaecologist. Those trained during Phase 1 plus 2 additional midwives participated in a 5-day workshop and pilot period, after which revisions to the checklist were made prior to implementation. The checklist prompted specific assessments and associated reminders at the point of labour triage, such as identification of abnormal FHR, presentation, evidence of leaking membranes, maternal vital signs and gestational age assessment using last normal menstrual period (LNMP) and fundal height. It described cardinal clinical signs and symptoms to increase suspicion of high-risk conditions so that the midwife was guided to an appropriate management plan. Supplemental information contains the Phase 2 checklist, including the diagnostic criteria used to raise suspicion of these conditions. Midwives were asked to use the checklist when assessing the mother upon presentation, then complete the intake log to document diagnoses before birth.

For Phase 3 (October 2018 - June 2019), following clinical assessments of the checklist, midwives additionally performed limited obstetric ultrasound to assess FHR, head position, placenta location, multiple gestation, deepest vertical fluid pocket, and biometry measures (biparietal diameter, head circumference, and femur length). The Phase 3 checklist, including the clinical and ultrasound diagnostic criteria related to the conditions of interest, is provided in Supplemental information. Midwives were asked to use the checklist and conduct the scan when assessing the mother, then complete the intake log to document intake diagnoses. Although ultrasound can be used to detect other conditions, such as foetal abnormalities, nuchal cord, foetal sex, etc., we focused on these measures because they can be identified after proper training and can assist in clinical decision-making during labour in resource-limited settings (Shah et al., 2011). As such, we refer to this triage scan protocol as limited obstetric ultrasound because it focused on a set of specific conditions that were of particular interest for this study. The ultrasound training curriculum was created by a team comprising in-country stakeholders and a U.S.-based expert in training ultrasonography in LMICs. A total of 7 midwives, 2 doctors and 2 research nurses received training over a 2-week period in October 2018. Robust quality assurance included 25 required proctored scans, requirement to pass an observed structured clinical exam, and all images produced 3 months post-training underwent blinded review to evaluate image quality and to flag common errors. These findings, as well as data regarding midwives' skill acquisition, confidence and perceptions of the ultrasound training course, are described elsewhere (Shah et al., 2020). Because of software defects in the ultrasound machines initially procured (SonoScape A5), data collected from October to December 2018 were excluded from the analysis due to inaccurate image measurements and interpretation. New ultrasound machines (Mindray DP-10) and a 1-hour refresher course were implemented in January 2019.

Outcomes after birth were determined in each phase by a standard outcome form, which relied on data abstraction from existing hospital data sources (the maternity register and patient medical charts). Midwives were also trained to complete a newborn assessment tool. For the conditions of interest, midwives used the following criteria to confirm presence of a complication at outcome: Multiple gestation was confirmed when more than one foetus was present. For preterm birth less than 37 weeks, midwives identify the infant in the maternity register as term or preterm based on standard practice clinical assessment. We also provided additional training to conduct the New Ballard exam and collect postnatal anthropometric measurements, such as birth weight, birth length and head circumference to help inform this designation (Ballard et al., 1991; Battaglia and Lubchenco, 1967). Providers reported oligohydramnios if there was reduced amniotic fluid at birth. Placenta praevia was diagnosed if vaginal bleeding or Caesarean section reported placenta praevia. Malpresentation was discerned if the presenting foetal part was non-cephalic (e.g. breech, transverse, oblique). Midwives assessed Apgar and breathing at birth as indications of probable abnormal FHR prior to birth. Intrauterine demise or intrapartum stillbirth was included in this outcome category because it was possible that a foetus under distress died in utero after intake assessment, for example, due to delayed care.

### Participants

Women who presented to the labour unit after 28 weeks of gestation with regular, intermittent cramping pain were eligible. We excluded women with conditions that required immediate clinical intervention, for whom there would be no time to complete the study interventions; for example, women with an eclamptic seizure, severe antepartum haemorrhage, or imminent birth. We also excluded mothers who were admitted but not in labour (e.g., malaria in pregnancy), and those who did not consent for participation.

### Sample size

Using data extracted from the maternity register at the same hospital for a concurrent study^20^ from March 2016 to March 2017, we estimated that 20% of women presenting for labour had one or more of the conditions of interest. We conservatively assumed a complication prevalence of 17.5% and that 75% of the time, providers were already correctly identifying the conditions of interest. Using an α error of 0.05, 70% statistical power, and a 1-tailed test, to test a 25% increased rate of accurate detection compared to the baseline rate (i.e., 13.3% in Phase 1 vs. 16.4% in subsequent phases), we required 1225 births with known birth outcomes in each phase. We increased the sample to account for 5% missing data (e.g., missing outcomes, refusal to consent or data quality issues).

### Data collection and management

A research nurse identified eligible women upon arrival to the maternity ward and obtained informed written consent. Information, including maternal and foetal diagnoses at intake, was recorded in the intake log across the phases. Participants were assessed at a single timepoint by study-trained midwives to determine maternal and foetal well-being. Triage assessment and interventions used were dependant on the phase in which the woman was enrolled. Specifically, during Phase 1, existing standard of care assessments were used; in Phase 2, the triage checklist was added to the triage assessment procedures; in Phase 3, the checklist with limited obstetric ultrasound scan was implemented. For outcome data for both singleton and multiple gestation births, a research nurse extracted data from the woman's medical chart, the facility maternity register, and the newborn assessment tool. These outcome-related data sources were completed by a clinical provider. The intake log, checklists and outcome form were in English, while consent forms for women were translated into Lusoga, the local language.

Data were collected on paper forms, kept in locked file cabinets in a secure location, and transferred via tablet to Open Data Kit (ODK), an open-source software designed for collecting and managing data in resource-limited contexts. Before data entry, study research nurses ensured consistent and non-duplicative study identification numbers. The study data manager also spot-checked data consistency between paper forms and ODK. Attainment of intended sample size was monitored by the study team during each phase. Specifically, every two weeks, the team counted the number of outcome forms that could be linked to intake data, as well as the woman's consent form. Lastly, aggregate counts of facility admissions, deliveries by Caesarean, live births, birthweight <2500 g and stillbirths were abstracted monthly from the maternity register to better understand enrolment rates and facility trends.

Data were converted into SPSS Version 25.02 (Armonk, NY: IBM Corp.) for cleaning, range and logic checks prior to analysis. All devices used for data entry or analyses were encrypted and password protected. All electronic data were maintained on secure systems with access limited to designated study staff, including the ODK server which is securely hosted by UC San Francisco.

### Data analysis

All individual-level data from the intake log, Phase 2 checklist, Phase 3 checklist and limited obstetric ultrasound, and outcome forms were linked through an individual study identification number and inpatient number. Our primary analysis was intent-to-treat (all women who consented at intake and had an outcome), as well as a per-protocol analysis (those who received all study components relevant to their study phase) for the composite variable (i.e. any condition).

To evaluate the comparability of study participants' sociodemographic and reproductive health characteristics, bivariate analyses stratified by phase were conducted using chi-square tests (with Fisher's exact statistics when any cell had less than 5 observations) for categorical data and Student's *t*-test for continuous data. Logistic regression with robust variance estimation analyses with adjustment for maternal age, education level, fuel source, attendance to 4 ANC, gestational age at intake, nulliparity, history of Caesarean/stillbirth/neonatal death, and infant sex were conducted.

To ensure that changes in diagnostic accuracy were not detrimental, we also evaluated phase differences in the rates of adverse birth outcomes, including 5-minute Apgar scores <7, intrapartum stillbirth, pre-discharge mortality and maternal mortality.

The study was originally powered using a one-tailed test. However, because the effect on the primary outcome was considerably larger than expected, we conservatively used and present two-tailed test statistics.

### Ethical considerations

All participants provided written informed consent. Ethical approvals were obtained from the Institutional Review Board at the University of California San Francisco (#17-23310), the Higher Degrees, Research and Ethics Committee at Makerere University in Uganda (#515) and the Uganda National Council for Science and Technology (#HS 2347).

## Results

**Fig. 1:**
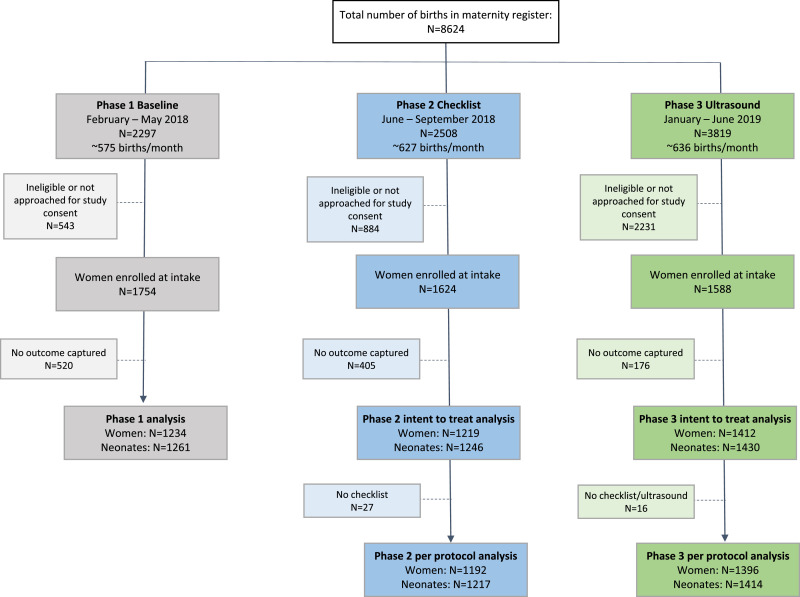
Participant flowchart by phase.

Between February 2018 and June 2019, we enrolled 76.4%, 64.8% and 41.6% of all deliveries in Phase 1, 2, and 3, respectively (Fig. 1). Missing outcome data ranged from 29.6% in Phase 1 to 11.1% in Phase 3. Therefore, a total of 3865 women and 3937 newborns with similar sample sizes per phase were included in the analysis. Most women enrolled in Phase 2 and 3 received the intervention, defined as having a completed intake, checklist, or checklist plus scan.

Most women were less than 35 years old (24.6 ± 5.58). The mean gestational age at intake was 38.6 ± 2.12 weeks (Table 1). Statistically significant differences in maternal education level, source of fuel and obstetric history were observed across the phases. These differences were incorporated into the adjusted analyses.

**Table 1:** Demographic characteristics and obstetric history amongst study participants by phase.

Maternal characteristics (*n* = 3865)	Phase 1		Phase 2		Phase 3		p-value across phases
	*n* = 1234	%	*n* = 1219	%	*n* = 1412	%	
**Maternal age**							
<20 years	245	19.9%	228	18.7%	291	20.6%	0.390
20–35 years	903	73.2%	900	73.8%	1039	73.6%	
>35 years	86	7.0%	91	7.5%	82	5.8%	
Maternal age (mean, SD)	24.5 (5.63)	24.8 (5.63)	24.5 (5.51)	0.274			
**Education level**							
None	7	0.6%	0	0.0%	10	0.7%	<0.001*
Some/completed primary	595	49.2%	555	45.6%	573	40.8%	
Some/completed secondary	572	47.3%	643	52.8%	754	53.6%	
Some/completed university	36	3.0%	20	1.6%	69	4.9%	
**Fuel source**							
Firewood	375	30.5%	565	46.3%	374	26.5%	<0.001*
Charcoal	852	69.3%	652	53.5%	1034	73.3%	
Gas/Electricity/Paraffin	3	0.2%	2	0.2%	3	0.2%	
Attended >=4 ANC visits	535	43.4%	465	38.3%	1024	72.7%	<0.001*
Nulliparous	428	34.7%	407	33.4%	535	37.9%	0.043*
History Caesarean section	116	9.6%	84	6.9%	143	10.3%	0.007*
History stillbirth	28	2.3%	25	2.1%	15	1.1%	0.075
History neonatal death	26	2.1%	22	1.8%	22	1.7%	0.705
Gestational age at intake (mean, SD)	38.5 (2.01)		38.8 (2.15)		38.6 (2.17)		0.003

Newborn characteristics (*n* = 3937)	Phase 1		Phase 2		Phase 3		p-value across phases
	*n* = 1261	%	*n* = 1246	%	*n* = 1430	%	
Infant sex (male)	622	50.4%	635	51.9%	760	53.6%	0.258
Birthweight <2.5kg	129	10.2%	126	10.1%	140	9.8%	0.925
**Monthly facility characteristics (data abstracted from maternity register)**							
Delivery volume (mean, SD)	575 (22.1)	627(29.7)	636 (27.1)	0.011			
% low birth weight (mean, SD)	14.42% (2.84)	12.46% (1.59)	13.79% (1.57)	0.396			
% stillbirth (mean, SD)	5.25% (1.39)	5.06% (0.6)	3.43% (1.17)	0.046			

n are the total number of women/newborns enrolled in each phase. Pearson Chi-Squared tests for proportions; Student's *t*-test for means.

The incidence of the maternal and foetal conditions of interest, as defined by outcomes after birth, was similar across the phases around 18% (Table 2). Preterm birth was the most common, 9.0%, 8.9% and 10.3% across the phases (*p* = 0.374). The incidence of other maternal conditions was <2.5%. No statistically significant differences in foetal conditions were observed. Foetal conditions were more common amongst multiple gestation births. amongst them, malpresentation increased across the phases (20.4%, 30.0% and 48.8%, *p* = 0.011).

**Table 2:** Incidence of conditions of interest amongst study participants by phase.

	Phase 1		Phase 2		Phase 3		p-value across phases
	n cases	%	n cases	%	n cases	%	
**Maternal conditions amongst all deliveries (*n*** **=** **3865)**							
Multiple gestation	27	2.2%	23	1.9%	25	1.8%	0.730
Oligohydramnios	28	2.3%	20	1.6%	18	1.3%	0.140
Placenta praevia	14	1.1%	8	0.7%	9	0.6%	0.283
Preterm birth	111	9.0%	109	8.9%	146	10.3%	0.374
Any maternal condition (composite)	155	12.6%	146	12.0%	178	12.6%	0.867
**Foetal conditions amongst all births (*n*** **=** **3937)**							
Malpresentation	38	3.0%	50	4.0%	54	3.8%	0.371
Abnormal foetal heart rate	71	5.6%	47	3.8%	64	4.5%	0.081
Any foetal condition (composite)	104	8.2%	93	7.5%	113	7.9%	0.766
*Any maternal or foetal condition (composite)*	233	18.5%	224	18.0%	264	18.5%	0.934

Any maternal condition includes multiple gestation, oligohydramnios, placenta praevia and preterm birth. Any foetal condition includes malpresentation and abnormal foetal heart rate. n cases are the number of outcome-defined cases. Pearson Chi-Squared tests for proportions; *significant at the 0.05 level.

Overall, the joint correct identification of cases and non-cases for the primary composite outcome was high and similar across the phases (*85.1%, 85.2 and 83.4%, p* *=* 0.339), since non-cases comprised the majority of the sample.

Sensitivity for the primary composite outcome increased from 47.0% in Phase 1, to 68.8% in Phase 2, and 73.5% in Phase 3 (Table 3, *p* < 0.001), indicating that more outcome-confirmed cases were correctly identified at labour triage intake. PPV declined in both Phase 2 and Phase 3 (55% and 48.7%, respectively), compared to Phase 1 (65.9%), indicating more false positives were diagnosed at intake with introduction of the interventions.

**Table 3:** Diagnostic sensitivity and positive predictive value by phase.

Sensitivity	n_1_ cases			% Sensitivity			Sensitivity p-values		
	*Ph1*	*Ph2*	*Ph3*	*Ph1*	*Ph2*	*Ph3*	*across phases*	*Ph2* vs*. 1*	*Ph3* vs*. 1*
Multiple gestation	21	17	23	77.8	73.9	92.0			
Oligohydramnios	9	11	14	32.1	55.0	77.8			
Placenta praevia	1	1	6	7.1	12.5	66.7			
Preterm birth	79	99	126	71.2	90.8	86.3			
Malpresentation	14	36	47	36.8	72.0	87.0			
Abnormal foetal heart rate	21	21	28	29.6	44.7	43.8			
Any maternal condition (composite)	88	115	150	56.8	78.8	84.3	<0.001*	<0.001*	<0.001*
Any foetal condition (composite)	33	54	70	31.7	58.1	61.9	<0.001*	<0.001*	<0.001*
*Any maternal or foetal condition (composite)*	119	154	194	47.0	68.8	73.5	<0.001*	<0.001*	<0.001*

Positive predictive value	n_2_ cases			% PPV			PPV p-values		
	*Ph1*	*Ph2*	*Ph3*	*Ph1*	*Ph2*	*Ph3*	*across phases*	*Ph2* vs*. 1*	*Ph3* vs*. 1*
Multiple gestation	21	17	23	87.5	63.0	88.5			
Oligohydramnios	9	11	14	52.9	37.9	18.7			
Placenta praevia	1	1	6	33.3	25.0	75.0			
Preterm birth	79	99	126	68.1	67.8	58.9			
Malpresentation	14	36	47	82.4	48.0	69.1			
Abnormal foetal heart rate	21	21	28	51.2	48.8	57.1			
Any maternal condition (composite)	96	117	137	66.2	61.6	47.6	<0.001*	0.383	<0.001*
Any foetal condition (composite)	35	54	70	60.3	48.2	63.6	0.056	0.133	0.675
*Any maternal or foetal condition (composite)*	143	148	167	65.9	55.0	48.7	<0.001*	0.015*	<0.001*

Any maternal condition includes multiple gestation, oligohydramnios, placenta praevia and preterm birth. Any foetal condition includes malpresentation and abnormal foetal heart rate. n_1_ equals the number of cases (true positives); for composite variables, outcome data are used to define non-mutually exclusive diagnoses. n_2_ equals the number of cases (true positives); for composite variables, intake data are used to define non-mutually exclusive diagnoses. *Chi-square statistic is significant at the 0.05 level. ^a^Fisher's exact test.

To ensure that decreased PPV, which is often associated with increased sensitivity, was not detrimental, we also evaluated phase differences in the rates of adverse maternal or foetal outcomes. Lower PPVs were not associated with differences in intrapartum stillbirth, pre-discharge maternal or neonatal deaths (Supplemental Table 1).

Adjustment for differences in sample characteristics across study phases did not obviate the improvements in sensitivity and reductions in PPV for the composite variables (Table 4). Per protocol analyses yielded nearly identical results given the small percentage of missing checklists/scans in Phase 2 and 3 (*data not shown*). Specifically, for the primary outcome composite variable, substantial and significant improvements in sensitivity and significant reductions in PPV were observed from Phase 1 to Phase 3.

**Table 4:** Logistic regression of sensitivity and positive predictive value controlled for covariates.

		Sensitivity				Positive predictive value			
		OR	95 C.I.		p-value	OR	95 C.I.		p-value
Any maternal condition (*n* = 3865)	*Phase number 1 (ref)*								
	Phase 2	2.19	1.18	4.08	0.013	0.80	0.49	1.29	0.354
	Phase 3	3.28	1.76	6.11	<0.001*	0.54	0.35	0.85	0.008
Any foetal condition (*n* = 3937)	*Phase number 1 (ref)*								
	Phase 2	2.99	1.63	5.47	<0.001*	0.66	0.33	1.30	0.226
	Phase 3	3.41	1.90	6.11	<0.001*	1.26	0.64	2.50	0.500
Any condition (*n* = 3937)	*Phase number 1 (ref)*								
	Phase 2	2.28	1.51	3.46	<0.001*	0.58	0.40	0.85	0.005
	Phase 3	2.58	1.72	3.87	<0.001*	0.47	0.33	0.67	<0.001*

Any maternal condition includes multiple gestation, oligohydramnios, placenta praevia and preterm birth. Any foetal condition includes malpresentation and abnormal foetal heart rate. Controlled for covariates: maternal age, education level, fuel source, attendance to 4ANC, gestational age at intake, nulliparity, history of Caesarean section. Additional models incorporating infant sex and history of stillbirth/neonatal death showed similar results (data not shown).

OR = odds ratio; CI = confidence interval (with lower and upper limits shown).

Given the subjective nature of assessment of oligohydramnios after birth without the use of ultrasound (Sherer, 2002; Figueroa et al., 2020), and the inconsistent assessment or documentation of premature rupture of membranes, we conducted *post hoc* unadjusted analysis excluding oligohydramnios from the composite outcome. Both the increases in sensitivity and reductions in PPV remained statistically significant (Supplemental Table 2).

## Discussion

This quasi-experimental study demonstrates that a triage checklist and limited obstetric ultrasound performed at point-of-care by midwives in a general hospital in a low-resource setting improved sensitivity for a composite measure of clinically important maternal and foetal conditions. Unsurprisingly, the joint correct identification of cases and non-cases remained high given the fairly low incidence of these conditions and that non-cases comprised the majority of the study sample. Therefore, we purposefully focused on clinically relevant attributes - sensitivity and PPV, which indicate the percent of cases that are identified or missed. In analyses adjusting for differences in sample characteristics, we found the magnitude of the improvements in Phase 2 and Phase 3 sensitivity for any maternal or foetal condition was 2—3 times greater than Phase 1 with some reduction in PPV.

This study raised interesting points about the potential utility of midwife-performed limited obstetric ultrasound for specific conditions in this context. Phase 3 exhibited fewer false positives than Phase 2 for malpresentation, multiple gestation, and praevia, suggesting improved detection of head position, foetal number and placental location. These results align with a previous study in Uganda whereby midwife-performed third trimester sonography corrected clinical diagnoses of foetal presentation and multiple versus singleton gestation (Swanson et al., 2014). On the other hand, increased false positives for preterm birth was observed in Phase 3, coupled with less sensitivity than Phase 2. Introduction of biometry measures in late pregnancy may have increased uncertainty of gestational age, which is consistent with wider margins of error for gestational age by ultrasound after 24 weeks (Papageorghiou et al., 2016). While interpretation about the individual conditions should be taken with caution given that the study was powered to test the composite measure, these data indicate that a truncated scan focusing on placental location, foetal position and number, may be relevant in this context.

This study explored both a low-tech intervention (checklist) and a high-tech intervention (limited obstetric ultrasound) in a single low-resource district hospital. Although the sample was small to detect differences, we observed no harm to maternal or perinatal outcomes associated with the interventions' increase in false positives, suggesting the potential for these interventions to safely improve labour triage practices especially in contexts where prior ANC has not been robust or well documented. We used a pre-post temporal comparison group, rather than a contemporaneous group, but logistic regression analyses adjusted for differences in study group characteristics to minimise this design limitation.

This study had several limitations. First, midwives who were trained to deliver the interventions also documented diagnosis at outcome, potentially introducing diagnostic (suspicion) bias over time. Second, we do not know the accuracy of reported outcomes after birth, some of which are often inaccurate, such as prematurity and oligohydramnios; conversely, some diagnoses may have been captured in prior ANC visits which may have informed intake data, but we did not systematically capture ANC information. Third, our eligibility criteria excluded women requiring immediate clinical intervention which could have affected inclusion of women with conditions or interest or women at higher risk of poor outcomes. Lastly, the study was conducted over a 16-month period and we cannot exclude the possibility that external factors did not influence our findings. For example, parallel efforts to enhance institutional births or facility quality of care cannot be accounted for.

We observed different enrolment rates and proportions of missing outcome data across the phases although similar sample sizes were attained across all phases. The drop in Phase 3 enrolment could be due to fewer eligible women or those willing to consent, which could have introduced selection bias. We noted demographic differences such as higher education, history of prior Caesarean section, and completion of four or more ANC visits. This could indicate, for example, that Phase 3 participants had more positive health-seeking behaviours, were more likely to come to the hospital early, or consent for a scan. However, in analyses that adjusted for differences in sample characteristics across study phases, improvements in sensitivity and reductions in PPV for the composite variables remained statistically significant, reflecting internal validity. Moreover, since a subset of midwives were trained in study procedures rather than all midwives on staff at the hospital, workload likely affected enrolment numbers and may have influenced selectivity of participants based on provider subjectivity or convenience. While we intended for each shift to be covered by at least one study-trained midwife, competing responsibilities and staff rotations/turnover were challenges in Phase 3 study implementation as evidenced by qualitative interviews exploring midwives' experience with triage ultrasound (manuscript submitted). These implementation challenges may point to the utility of targeted scanning of women based on initial risk or symptom assessment versus universal scanning. Other challenges emerged, such as power outages, machine battery life, overheating, etc. which are consistent with other LMIC ultrasound studies (Kim et al., 2018). While these data limit study generalizability, they reflect key considerations for real-world implementation and focus on effectiveness as opposed to testing the interventions under falsely ideal conditions.

## Conclusion

This study reveals that a triage checklist and limited obstetric ultrasound administered by midwives improved correct case identification of a composite measure of maternal and foetal conditions of interest. These interventions merit further evaluation to ascertain their cost-effectiveness and utility in other settings, as well as impact on maternal and newborn outcomes. Furthermore, modifications such as targeted scanning of women based on risk or symptoms, implementation of a truncated scan protocol which focuses on select conditions, or the addition of dedicated facility staff, warrant consideration.

## Data availability

The datasets generated and/or analysed for the current study are available from the corresponding author upon reasonable request.

## Ethical approval

All participants provided written informed consent. Ethical approval for this study was obtained from the Institutional Review Board at the University of California San Francisco (#17–23310), the Higher Degrees, Research and Ethics Committee at Makerere University in Uganda (#515) and the Uganda National Council for Science and Technology (#HS 2347). Initial approvals were obtained October 28, 2017, November 7, 2017, and January 19, 2018, respectively, and renewed annually.

## Funding sources

This study was supported by the East Africa Preterm Birth Initiative (PTBi-EA), a multi-year, multi-country effort generously funded by the 10.13039/100000865Bill & Melinda Gates Foundation (OPP1107312), Seattle, WA.

## Clinical trial registry

Not applicable

## CRediT authorship contribution statement

**Jude Mulowooza:** Conceptualization, Writing – original draft, Writing – review & editing, Project administration. **Nicole Santos:** Conceptualization, Methodology, Formal analysis, Data curation, Writing – original draft, Writing – review & editing, Project administration. **Nathan Isabirye:** Data curation, Project administration, Writing – review & editing. **Innocent Inhensiko:** Software, Data curation, Project administration. **Nancy L. Sloan:** Conceptualization, Methodology, Formal analysis, Writing – original draft, Writing – review & editing. **Sachita Shah:** Methodology, Writing – review & editing, Resources. **Elizabeth Butrick:** Conceptualization, Methodology, Writing – review & editing. **Peter Waiswa:** Conceptualization, Writing – review & editing, Supervision, Project administration. **Dilys Walker:** Funding acquisition, Conceptualization, Methodology, Supervision.

## Declaration of Competing Interest

The authors of this study have no financial conflicts of interest to report.

## Appendix Supplementary materials

Image, application 1
