# Supplementary material for: Midwife-performed checklist and ultrasound to identify obstetric conditions at labour triage in Uganda: A quasi-experimental study
Source: Midwifery. 2021 May;96:102949. doi: 10.1016/j.midw.2021.102949 (PMC7988503; doi:10.1016/j.midw.2021.102949)
Supplement: Supplementary file 1 [file mmc1.docx]

**Table S1.** Secondary outcomes among all study participants across phases.

| **All study participants** | **Phase 1** | | **Phase 2** | | **Phase 3** | | **across Ph** | **Ph2 vs. 1** | **Ph3 vs. 1** |
| --- | --- | --- | --- | --- | --- | --- | --- | --- | --- |
|  | **n** | **%** | **n** | **%** | **n** | **%** | **p-value** | **p-value** | **p-value** |
| Mode of delivery by caesarean^§^ | 277 | 22.5% | 305 | 25.1% | 383 | 27.2% | 0.019 | 0.134 | 0.005 |
| Maternal death pre-discharge | 4 | 0.3% | 2 | 0.2% | 1 | 0.1% | 0.308 | 0.687^a^ | 0.192^a^ |
| Apgar 5 <7 | 60 | 4.8% | 46 | 3.7% | 53 | 3.7% | 0.290 | 0.185 | 0.175 |
| Intrapartum stillbirth | 11 | 0.9% | 14 | 1.1% | 8 | 0.6% | 0.277 | 0.527 | 0.334 |
| Pre-discharge neonatal mortality | 4 | 0.3% | 1 | 0.1% | 3 | 0.2% | 0.419^a^ | 0.375^a^ | 0.713^a^ |

For all study participants, n=3865 for mode of delivery and maternal death; n=3937 for newborn outcomes. ^§^For women diagnosed with a condition at intake either correctly or incorrectly (n=802), mode of delivery by caesarean was 42% in Phase 1 (n=81); 31.8% in Phase 2 (n=85) and 38% in Phase 3 (n=130) with a p-value of 0.072. ^a^Fisher's exact test.

**Table S2:** Composite maternal and any maternal/foetal outcomes excluding oligohydramnios.

| **SENSITIVITY** | **Phase 1** | | **Phase 2** | | **Phase 3** | | **p-value across phases** |
| --- | --- | --- | --- | --- | --- | --- | --- |
|  | **n_1_ cases** | **%** | **n_1_ cases** | **%** | **n_1_ cases** | **%** |  |
| Any maternal condition (composite) | 85 | 63.4% | 107 | 82.3% | 141 | 84.9% | <0.001* |
| *Any maternal or foetal condition (composite)* | 100 | 53.8% | 139 | 72.0% | 177 | 75.6% | <0.001* |
| **POSITIVE PREDICATIVE VALUE** | **Phase 1** | | **Phase 2** | | **Phase 3** | | **p-value across phases** |
|  | **n_2_ cases** | **%** | **n_2_ cases** | **%** | **n_2_ cases** | **%** |  |
| Any maternal condition (composite) | 92 | 69.2% | 109 | 65.3% | 135 | 59.7% | 0.179 |
| *Any maternal or foetal condition (composite)* | 140 | 67.6% | 141 | 56.4% | 169 | 58.7% | 0.038* |

Any maternal condition includes multiple gestation, oligohydramnios, placenta praevia and preterm birth. Any foetal condition includes malpresentation and abnormal foetal heart rate. n_1_ equals the number of cases (true positives); for composite variables, outcome data are used to define non-mutually exclusive diagnoses. n_2_ equals the number of cases (true positives); for composite variables, intake data are used to define non-mutually exclusive diagnoses. *Chi-square statistic is significant at the 0.05 level.

**Supporting information.** Triage checklist, phase 2.

| **Facility Name** | **FIRST PRESENTATION OF THE MOTHER (TRIAGE)** | | | |
| --- | --- | --- | --- | --- |
| Mother’s Name: | | Age: | Date: | Time assessment  started: |
| Study ID #: | | | Gravidity: | Parity: |
| IP #: | | | Provider’s name: | |
| Last normal menstrual period (LNMP): __________________  Gestational age (GA) by LNMP: _______________________  GA by fundal height with tape measure, cm: ______________ | | | *If <37 weeks (preterm) and symptoms or signs of labour, admit or refer. Administer antenatal corticosteroids per national guideline if GA <34 weeks* | |
| Previous ultrasound this pregnancy? □ Yes □ No □ Unknown  Date of earliest previous ultrasound: _______________  GA at time of that ultrasound: _____weeks _______days  GA **today** by that ultrasound: _____weeks _______days | | |  |  |
| VS: BP ___________ Maternal HR _______ Temp __________ | | | - *If systolic blood pressure >140 or diastolic blood pressure >90, admit or refer. Start management of pre-eclampsia.* - *If >38°C or > 37.5°C axillary with signs of infection, admit or refer.* - *If maternal heart rate > 110 bpm, admit or refer.* | |
| Foetal HR __________ bpm  □ No foetal cardiac activity  Is there any clinical suspicion for **foetal** **distress**?  □ Yes □ No □ Not sure  Is there any clinical suspicion for **foetal demise**?  □ Yes □ No □ Not sure | | | - *If FHR is >160, admit and assess for signs of infection and monitor carefully.* - *If FHR <110, admit, provide intrauterine resuscitation, and monitor for fetal distress.* - *If no FHR, admit and plan for vaginal birth unless there is a maternal indication for Caesarean.* | |
| Multiple gestation □ No □ Yes  Is there any clinical suspicion for multiple gestation?  □ Yes □ No □ Not sure | | | *If multiple fetuses, monitor carefully throughout labour.* | |
| Presentation/Lie: □ Cephalic □ Breech □ Transverse / Oblique  Is there any clinical suspicion for non-cephalic presentation?  □ Yes □ No □ Not sure | | | *If non-cephalic presentation, consider referral and/or close monitoring in labour* | |
| Is there any clinical suspicion for placenta previa?  □ Yes □ No □ Not sure  Is there any clinical suspicion for oligohydramnios?  □ Yes □ No □ Not sure  Is there any clinical suspicion for ruptured membranes?  □ Yes □ No □ Not sure  Does the mother need to be admitted or referred for:  □ Heavy vaginal bleeding  □ Preterm labor or rupture of membranes  □ Active Labor  □ Delivery Imminent  □ Rupture of membranes  □ Other, specify: ___________________________ | | | - *If signs of placenta previa or abruption, admit or refer.* - *If preterm, initiate appropriate care or refer.* - *If dilation more than 4 cm with regular contractions, admit or refer*. - *If ruptured membranes, consider admission and antibiotic treatment if > 12 hours* | |
| Disposition:  □ Admitted  □ Referred. Specify referral location: ________________  □ Sent home  □ Not admitted, follow up at labour ward in _____ hours  □ Admitted for failed referral. Specify reason:  □ Transport problem  □ Time of day  □ Weather  □ Patient or family refusal  □ Other (specify): | | | *If unable to manage, refer. Reason for admission or referral (specify):* | |
| Time triage assessment completed: _____________ | | |  | |

**Supporting information.** Triage checklist plus limited obstetric ultrasound scan, phase 3.

| **Facility Name:** | **FIRST PRESENTATION OF THE MOTHER (TRIAGE)** | | | | | |
| --- | --- | --- | --- | --- | --- | --- |
| Mother’s Name: | | Age: | Date: | | Time assessment started: | |
| Study ID #: | | | Gravidity: | Parity: | | Prior scars: |
| IP #: | | | Provider’s name: | | | |
| Is the patient a referral from another facility?  □ Yes From where: _________________________  □ No  □ Not sure | | |  | | | |
| Last normal menstrual period (LNMP): __________________  Gestational age (GA) by LNMP: _______________________  GA by fundal height with tape measure, cm: ______________ | | | *If <37 weeks (preterm) and symptoms or signs of labour, admit or refer. Administer antenatal corticosteroids per national guideline if GA <34 weeks.* | | | |
| Previous ultrasound this pregnancy? □ Yes □ No □ Unknown  Date of earliest previous ultrasound: _______________  GA at time of that ultrasound: _____weeks _______days  GA **today** by that ultrasound: _____weeks _______days | | |  |  |  |  |
| Does patient have a history of preterm delivery?  □ Yes □ No | | | - *If yes, risk of preterm delivery is elevated.* | | | |
| Vital signs:  BP ___________ Maternal HR _______ Temp __________ | | | - *If systolic blood pressure >140 or diastolic blood pressure >90, admit or refer. Start management of pre-eclampsia.* - *If >38°C or > 37.5°C axillary with signs of infection, or maternal HR >110 admit or refer.* | | | |
| Is there clinical suspicion for **preterm labor** by any of the GA measures above?  □ Yes □ No □ Not sure | | |  | | | |
| Foetal HR by Foetoscope __________ bpm  □ No foetal cardiac activity  Is there any clinical suspicion for **foetal** **distress**?  □ Yes □ No □ Not sure  Is there any clinical suspicion for **foetal demise**?  □ Yes □ No □ Not sure | | | - *If FHR is >160, admit and assess for signs of infection and monitor carefully.* - *If FHR <120, admit, provide intrauterine resuscitation, and monitor for fetal distress.* - *If no FHR, admit and plan for vaginal birth unless there is a maternal indication for Caesarean.* | | | |
| **Presentation by palpation**:  □ Cephalic □ Breech □ Transverse / Oblique | | | *If non-cephalic presentation, consider referral and/or close monitoring in labour* | | | |
| Is there any clinical suspicion for **multiple gestation**?  □ Yes □ No □ Not sure | | | *Suspect multiple gestation if the fundal height is greater than the weeks of amenorrhea or if there is more than one FHR.* | | | |
| Is there any clinical suspicion for **placenta previa**?  □ Yes □ No □ Not sure | | | *Suspect placental previa if there is a history of PV bleeding accompanied by no abdominal pain.* | | | |
| Is there any clinical suspicion for **oligohydramnios**?  □ Yes □ No □ Not sure | | | *Suspect oligohydramnios if any of the following:*   - *the fundal height is much less than the weeks of amenorrhea* - *any fetal parts are easy to feel abdominally* - *any history of draining liquor* | | | |
| Is there any clinical suspicion for **ruptured membranes**?  □ Yes □ No □ Not sure | | | *If ruptured membranes, consider admission and antibiotic treatment if > 12 hours. DO NOT MEASURE Deepest Vertical Pocket.*  *Note: Scan may be difficult.* | | | |

| **ULTRASOUND FINDINGS TODAY** | | | |
| --- | --- | --- | --- |
| Fetal heart rate: __________ bpm  □ No foetal heart rate | | - *If NO or LOW FHR, STOP SCAN and plan for vaginal birth in the absence of indication for caesarean for IUFD or STAT CESAREAN for fetal distress.* - *If FHR is >160, admit and assess for signs of infection and monitor carefully. If FHR <120, admit, provide intrauterine resuscitation, and monitor for fetal distress.* | |
| Presentation: □ Cephalic □ Breech □ Transverse or Oblique | | *If non-cephalic, consider referral and/or close monitoring in labour.* | |
| BPD _______ cm □ unable to measure  HC _________ cm □ unable to measure  FL____________cm □ unable to measure    Average GA by US REPORT _____ weeks _____ days +/-3 wks  EDD___________ by US REPORT    CER/TCD ___________ cm □ unable to measure | | *If <37 weeks (preterm) with signs or symptoms of labour, admit and initiate appropriate care or refer. Administer antenatal corticosteroids per national guideline if <34 weeks.*    *Use REPORT for Average GA. If Report GA inaccurate or unavailable, use BPD for GA and indicate ERROR next to average GA. Measure CER/TCD AFTER REPORT AVERAGE GA.*    *Note: A known, reliable LMP or first trimester ultrasound will give more reliable dates than a third trimester ultrasound.* | |
| Multiple gestation: □ No □ Yes | | *If multiple fetuses, admit if in labor and monitor carefully. Continue this data sheet for the presenting fetus, and use an additional data sheet(s) for measures of additional fetuses by ultrasound. Attach all sheets together.* | |
| Placenta: (check all that apply)  □ Posterior □ Anterior □ Fundal □ Other__________ | | *If anterior placenta and history of caesarean, consider possibility of abnormal placentation (accreta).* | |
| Previa: □ No □ Yes *If Yes:* □ Complete □ Partial □ Unsure  Low Lying: □ No □ Yes | | *If placental previa and in labour/at term, admit and prepare for C-section, or refer.* | |
| Amniotic fluid deepest vertical pocket (DVP): __________ cm | | *Normal DVP is 2cm-8cm. Normal AFI is 8cm-24cm. If DVP <2cm, do full AFI before diagnosing with oligohydramnios. If DVP < 2cm AND AFI <8cm, consider oligohydramnios versus ruptured membranes and consider induction of labour if indicated. If DVP >8cm, consider polyhydramnios.* | |
| **MANAGEMENT PLAN** | | | |
| Does the mother have any of the diagnoses below?  □ No □ Yes *If Yes, select all that apply.* | | *If dilation more than 4 cm with regular contractions, admit or refer*. |  |
| □ Fetal demise  □ Fetal distress  □ Preterm labor  □ Multiple gestation  □ Malpresentation  □ Heavy vaginal bleeding | □ Oligohydramnios  □ Placenta previa  □ Rupture of membranes  □ Active Labor  □ Delivery Imminent  □ Other: ____________ |  |  |
| Disposition:  □ Admitted  □ Referred. Specify referral location: ________________  □ Sent home  □ Not admitted, follow up at labour ward in _____ hours  □ Admitted for failed referral. Specify reason:  □ Transport problem □ Weather  □ Time of day □ Patient or family refusal  □ Other (specify):______________________________ | | *If unable to manage, refer and indicate reason for referral.* |  |
| Did the ultrasound change your management plan today?  □ No  □ Yes | | If yes, please explain how the ultrasound changed your management: *(check all that apply)*  □ Suggest a cesarean section instead of vaginal birth  □ Suggest a vaginal birth instead of cesarean section  □ Plan for preterm delivery instead of term delivery  □ Refer to other facility instead of delivery at your facility  □ Other (specify): _____________________________ | |
| Time triage assessment completed: ____________ | |  | |
